# Supplementary material for: Intracellular Pocket Conformations Determine Signaling Efficacy through the μ Opioid Receptor
Source: J Chem Inf Model. 2025 Jan 17;65(3):1465–75. doi: 10.1021/acs.jcim.4c01437 (PMC11817682; doi:10.1021/acs.jcim.4c01437)
Supplement: Supplementary file 1 — ci4c01437_si_001.pdf [file ci4c01437_si_001.pdf]

Supporting Information for

Intracellular pocket conformations determine signaling efficacy through the μ opioid receptor

David A. Cooper,[1] Joseph DePaolo-Boisvert,[1] Stanley A. Nicholson,[2] Barien Gad,[2,3] David D. L. Minh[1,3]*
[1]Department of Chemistry, [2]Department of Applied Mathematics, [3]Department of Biology, Illinois Institute of Technology; Chicago, United States of America.
*Corresponding author: David D. L. Minh.

Email: dminh@iit.edu

This PDF file includes:

Figures S1 to S12
Tables S1 to S2
SI References

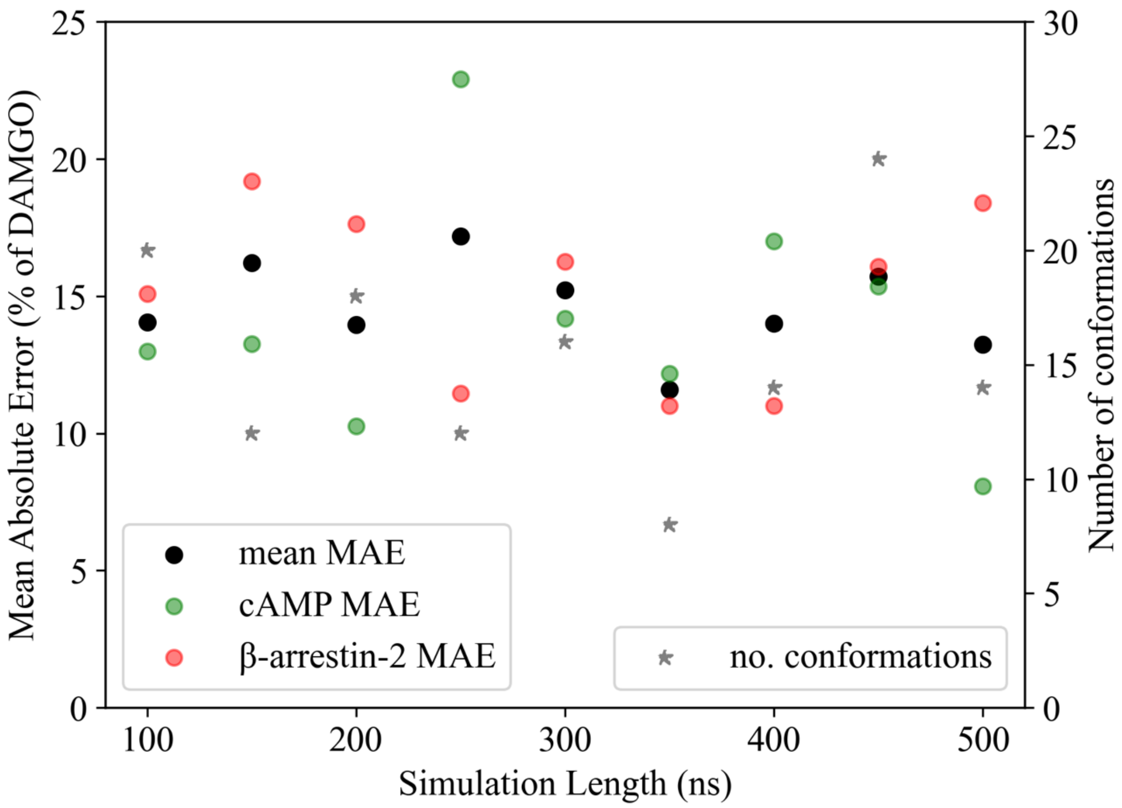

Figure S1 Model parameters and performance as a function of simulation length

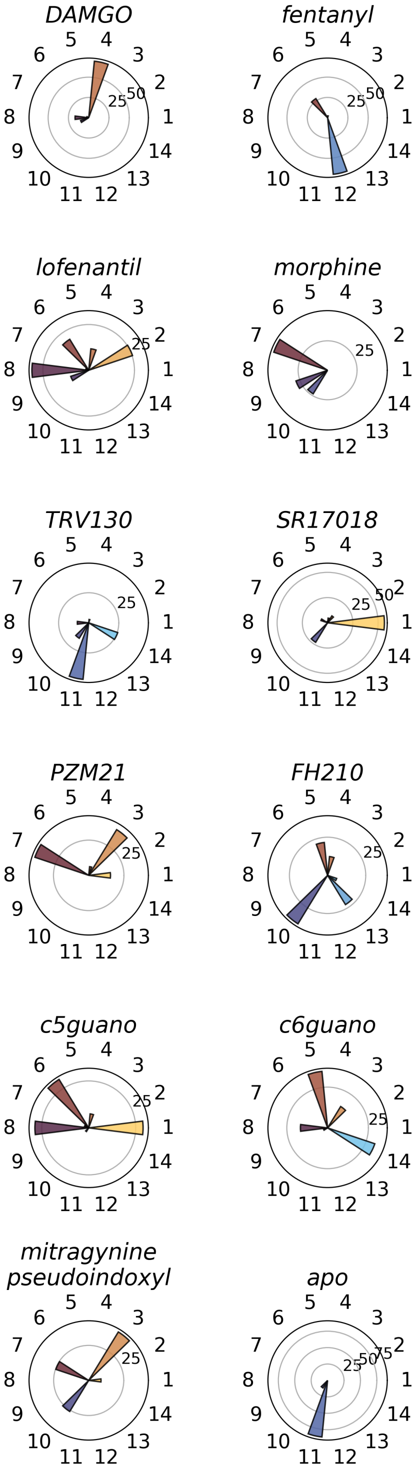

Figure S2 Percentages of simulations of complexes with different ligands in each conformation**.**

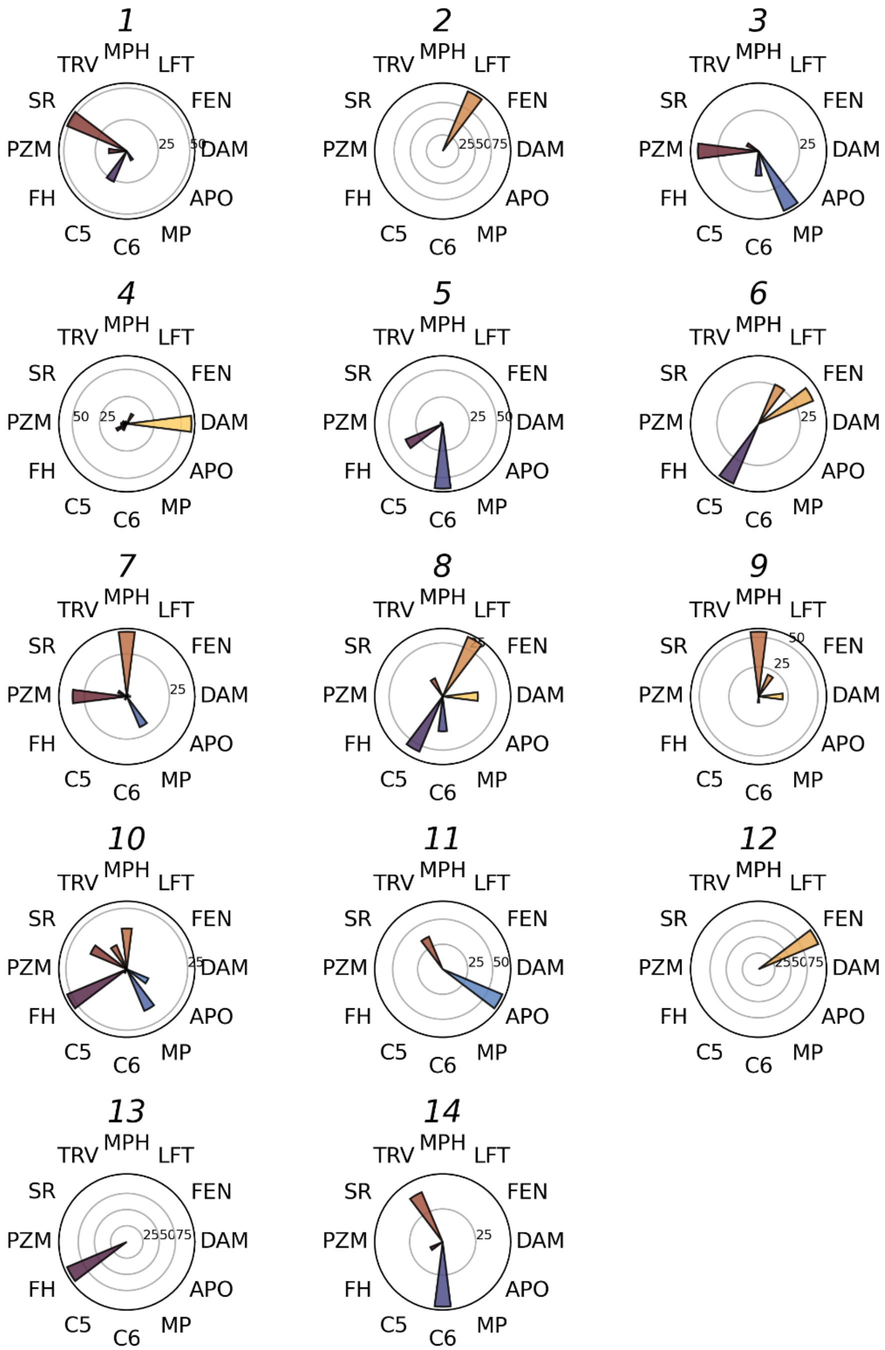

Figure S3 Percentages of conformations accessed in simulations with each complex. Abbreviations: DAM: DAMGO, FEN: fentanyl, LFT: lofentanil, MPH: morphine, TRV: TRV130, SR: SR17018, PZM: PZM21, FH: FH210, C5: c5guano, C6: c6guano, MP: mitragynine pseudoindoxyl, APO: apo.

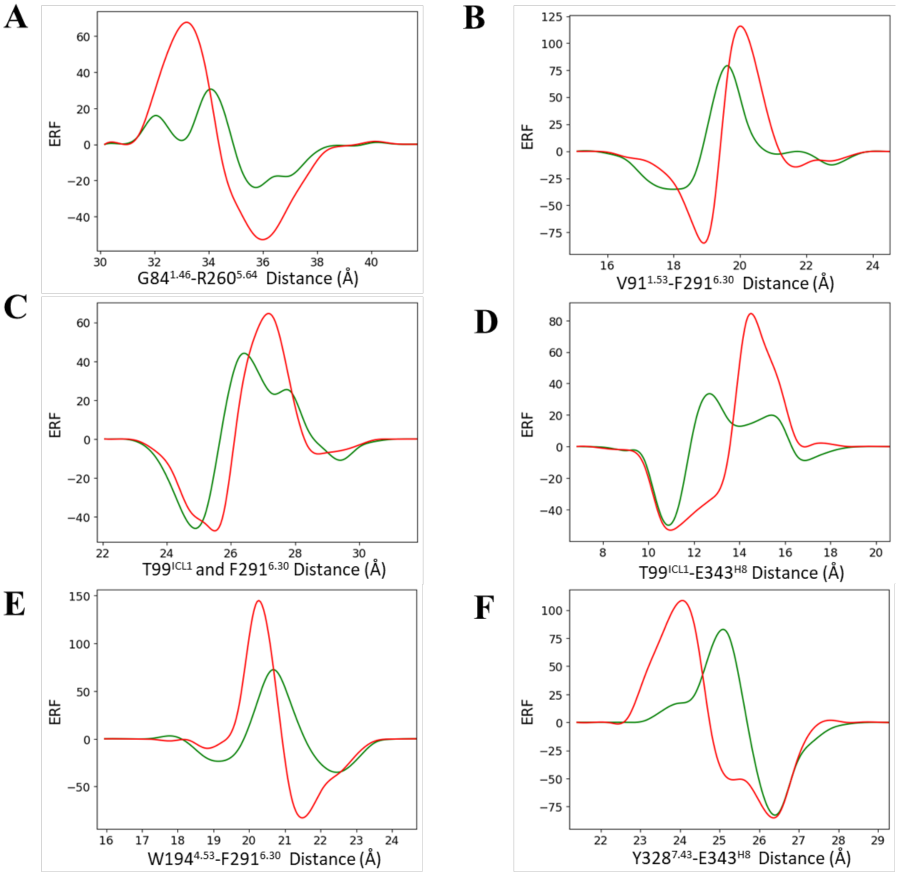

Figure S4 Efficacy response functions for alpha carbon distances with the largest general activation scores: (A) G84$^{1.46}$-R260$^{5.64}$, (B) V91$^{1.53}$-F291$^{6.30}$, (C) T99^{ICL1}-F291$^{6.30}$, (D) T99^{ICL1}-E343^{H8}, (E) W194$^{4.53}$-F291$^{6.30}$, and (F) Y328$^{7.43}$-E343^{H8}. The green and red curves correspond to the G protein and β-arrestin-2 efficacy response functions (ERF), respectively.

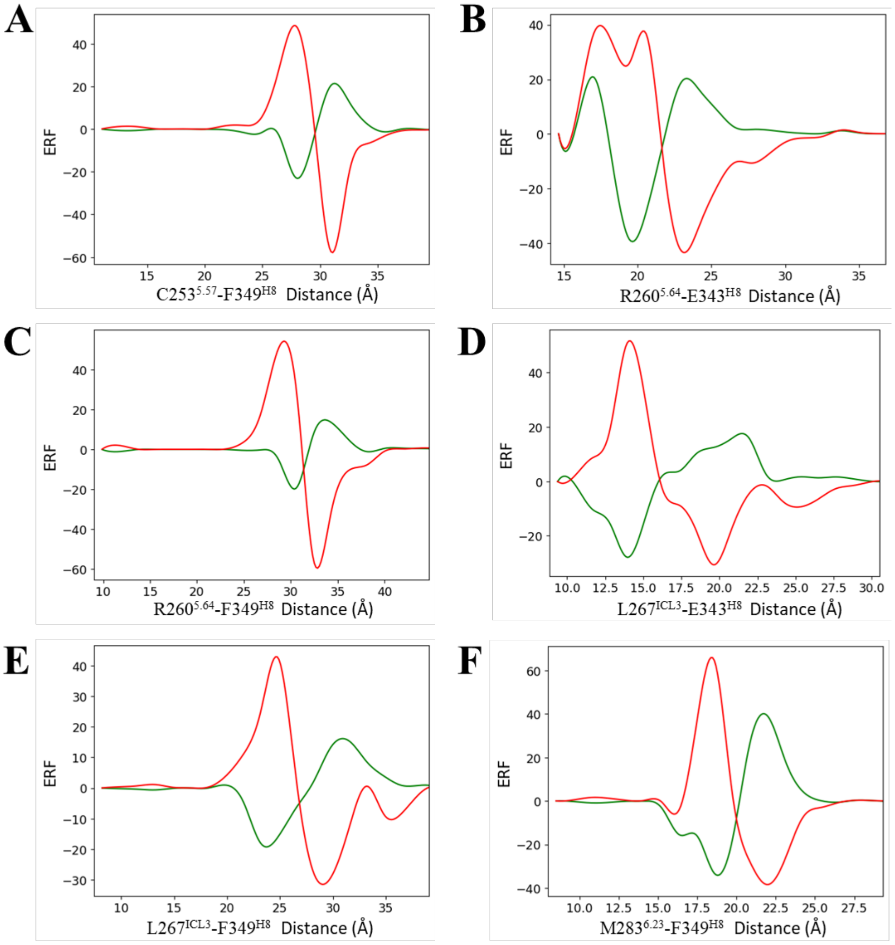

Figure S5 Efficacy response functions for alpha carbon distances with the largest selective activation scores: (A) C253$^{5.57}$-F349^{H8}, (B) R260$^{5.64}$-E343^{H8}, (C) R260$^{5.64}$-F349^{H8}, (D) L267^{ICL3}-E343^{H8}, (E) L267^{ICL3}-F349^{H8}, and (F) M283$^{6.23}$-F349^{H8}. The green and red curves correspond to the G protein and β-arrestin-2 efficacy response functions (ERF), respectively.

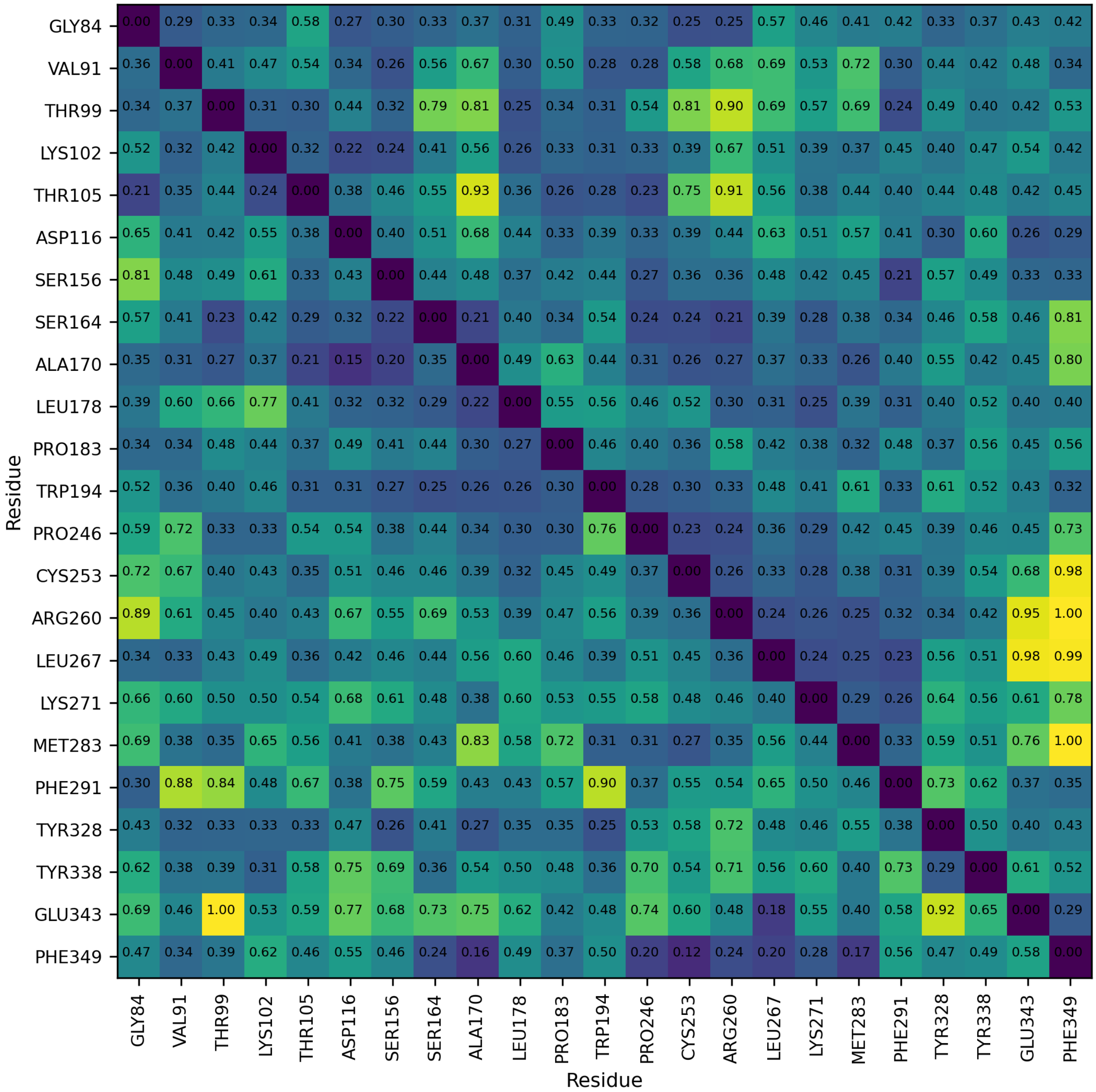

Figure S6 Efficacy response function scores for pairwise distances between alpha carbons from residues in the middle and intracellular end of each helix, with at least one residue in between, and the middle of each intracellular loop. Scores below the diagonal were calculated with the general activation function (Equation 8). Scores above the diagonal were calculated with the selective activation function (Equation 9). Scores from each category were divided by the largest score in the category.

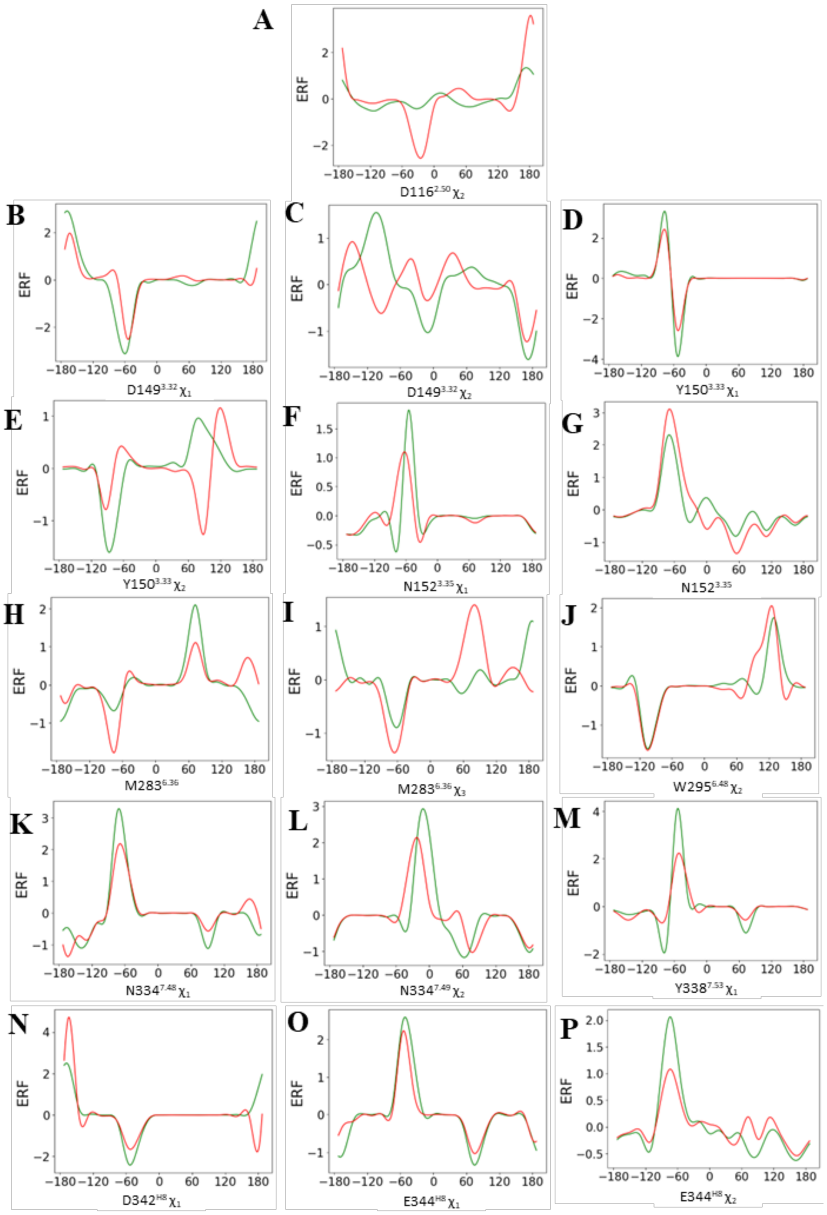

Figure S7 Efficacy response functions for dihedral angles with the largest general activation scores**:** **(A)** $D116^{2.50}$ χ_2 angle, **(B)** $D149^{3.32}$ χ_1 angle, **(C)** $D149^{3.32}$ χ_2 angle, **(D)** $Y150^{3.33}$ χ_1 angle, **(E)** $Y150^{3.33}$ χ_2 angle, **(F)** $N152^{3.35}$ χ_1 angle, **(G)** $N152^{3.35}$ χ_2 angle, **(H)** $M283^{6.36}$ χ_2 angle, **(I)** $M283^{6.36}$ χ_3 angle, **(J)** $W295^{6.48}$ χ_2 angle, **(K)** $N334^{7.48}$ χ_1 angle, **(L)** $N334^{7.49}$ χ_2 angle, **(M)** $Y338^{7.53}$ χ_1 angle, **(N)** $D342^{H8}$ χ_1 angle**, (O)** $E344^{H8}$ χ_1 angle, **(P)** $E344^{H8}$ χ_2 angle. The green and red curves correspond to the G protein and β-arrestin-2 efficacy response functions (ERF), respectively.

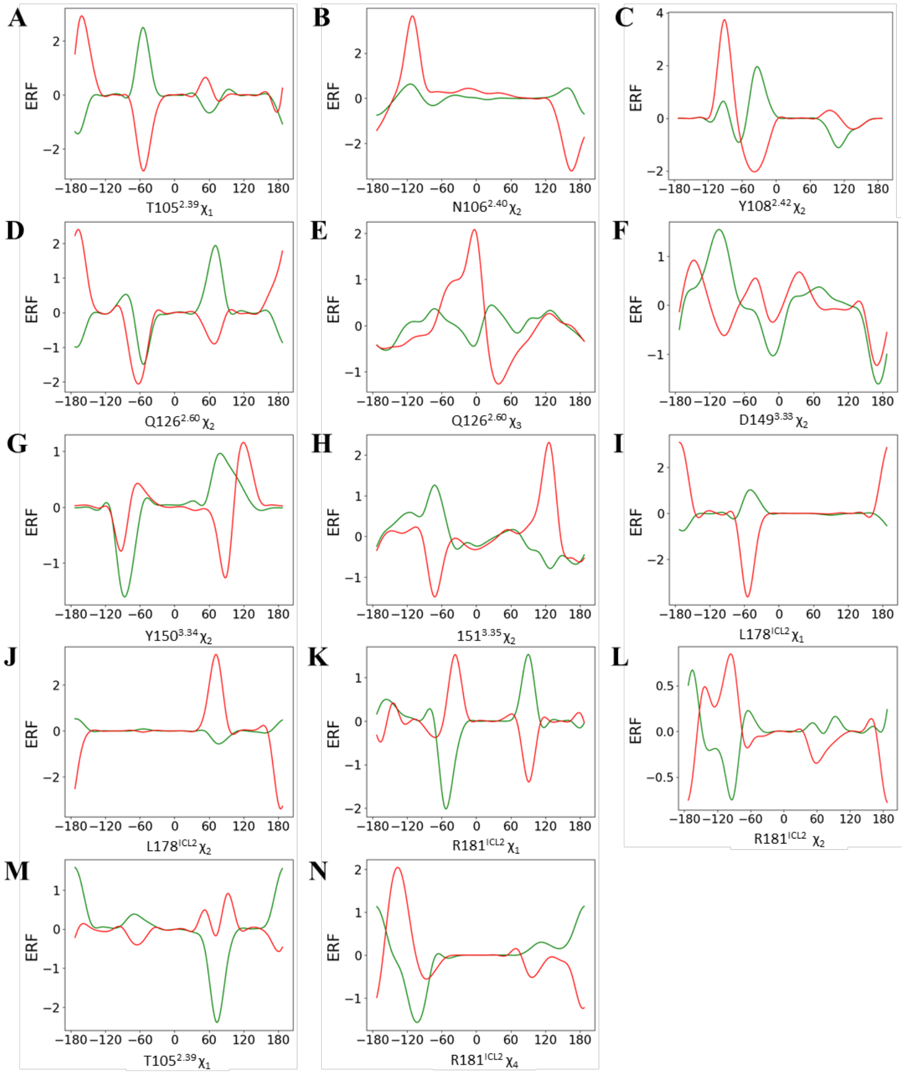

Figure S8 Efficacy response functions for dihedral angles with the largest selective activation scores: (A) T105$^{2.39}$ χ_1 angle, (B) N106$^{2.40}$ χ_2 angle, (C) Y108$^{2.42}$ χ_2 angle, (D) Q126$^{2.60}$ χ_2 angle, (E) Q126$^{2.60}$ χ_3 angle, (F) D149$^{3.32}$ χ_2 angle, (G) Y150$^{3.33}$ χ_2 angle, (H) 151$^{3.35}$ χ_2 angle, (I) L178^{ICL2} χ_1 angle, (J) L178^{ICL2} χ_2 angle, (K) R181^{ICL2} χ_1 angle, (L) R181^{ICL2} χ_2 angle, (M) R181^{ICL2} χ_3 angle, (N) R181^{ICL2} χ_4 angle. The green and red curves correspond to the G protein and β-arrestin-2 efficacy response functions (ERF), respectively.

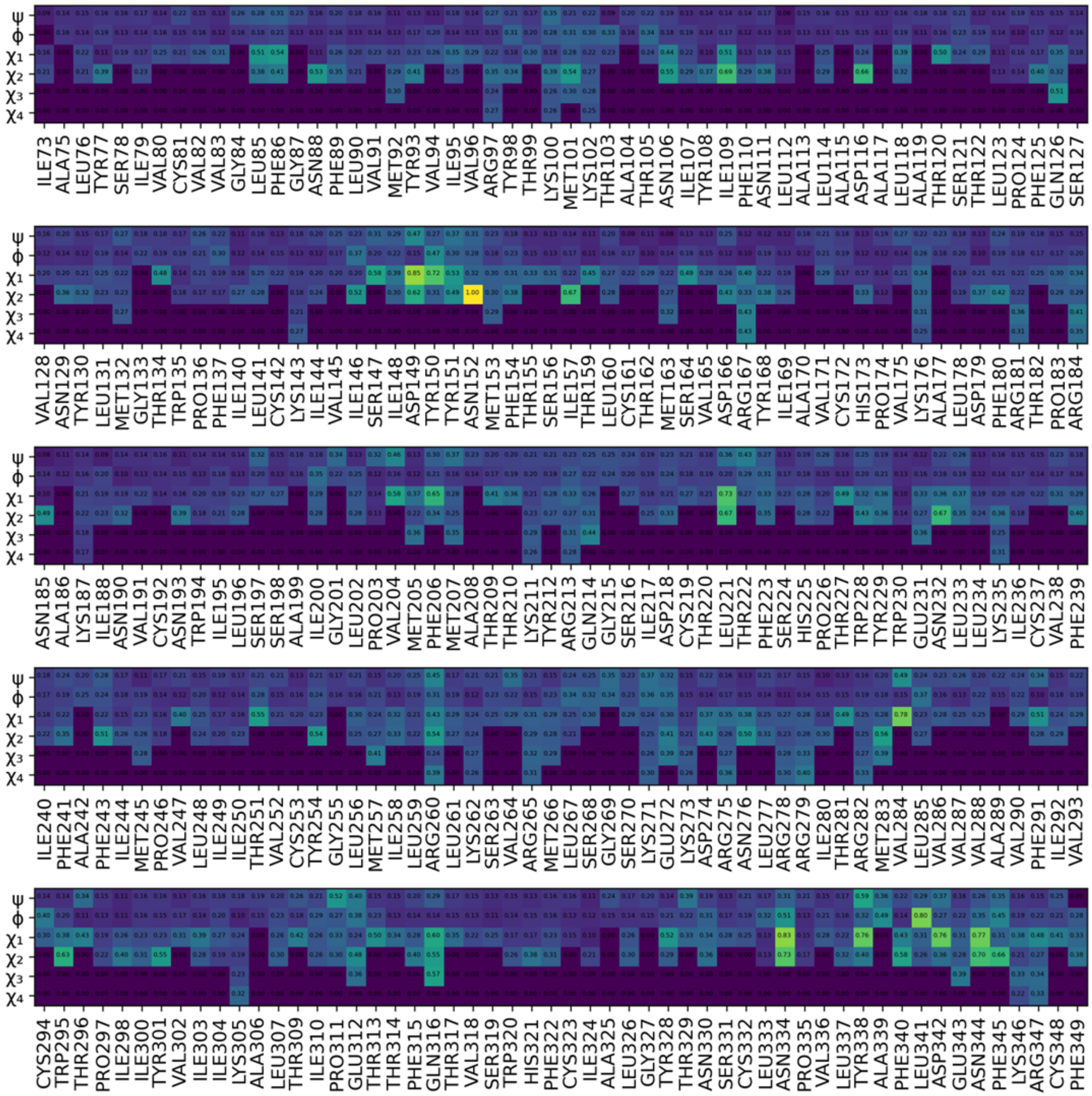

Figure S9 General activation function scores for amino acid dihedral angles.

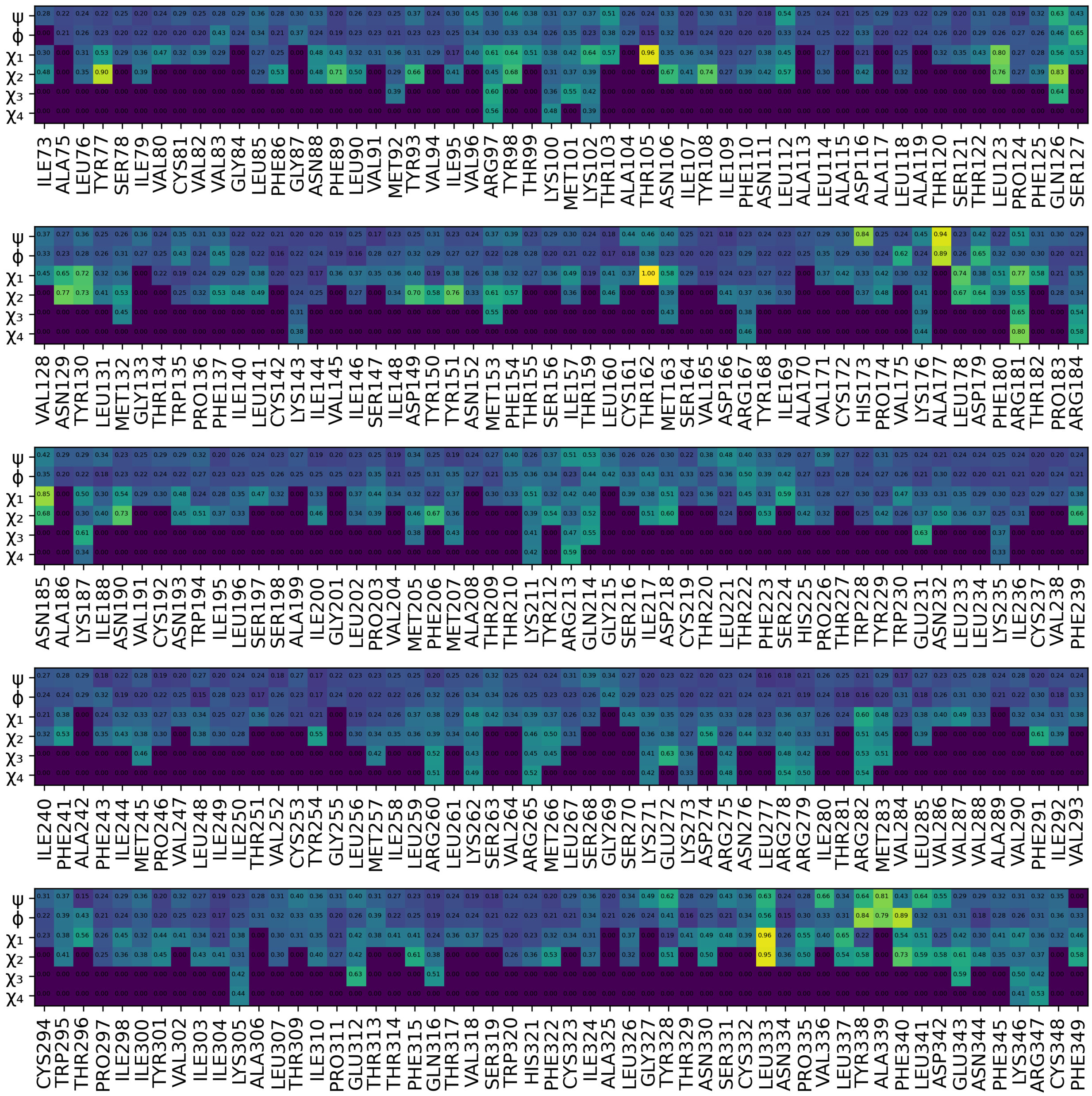

Figure S10 Selective activation function scores for amino acid dihedral angles.

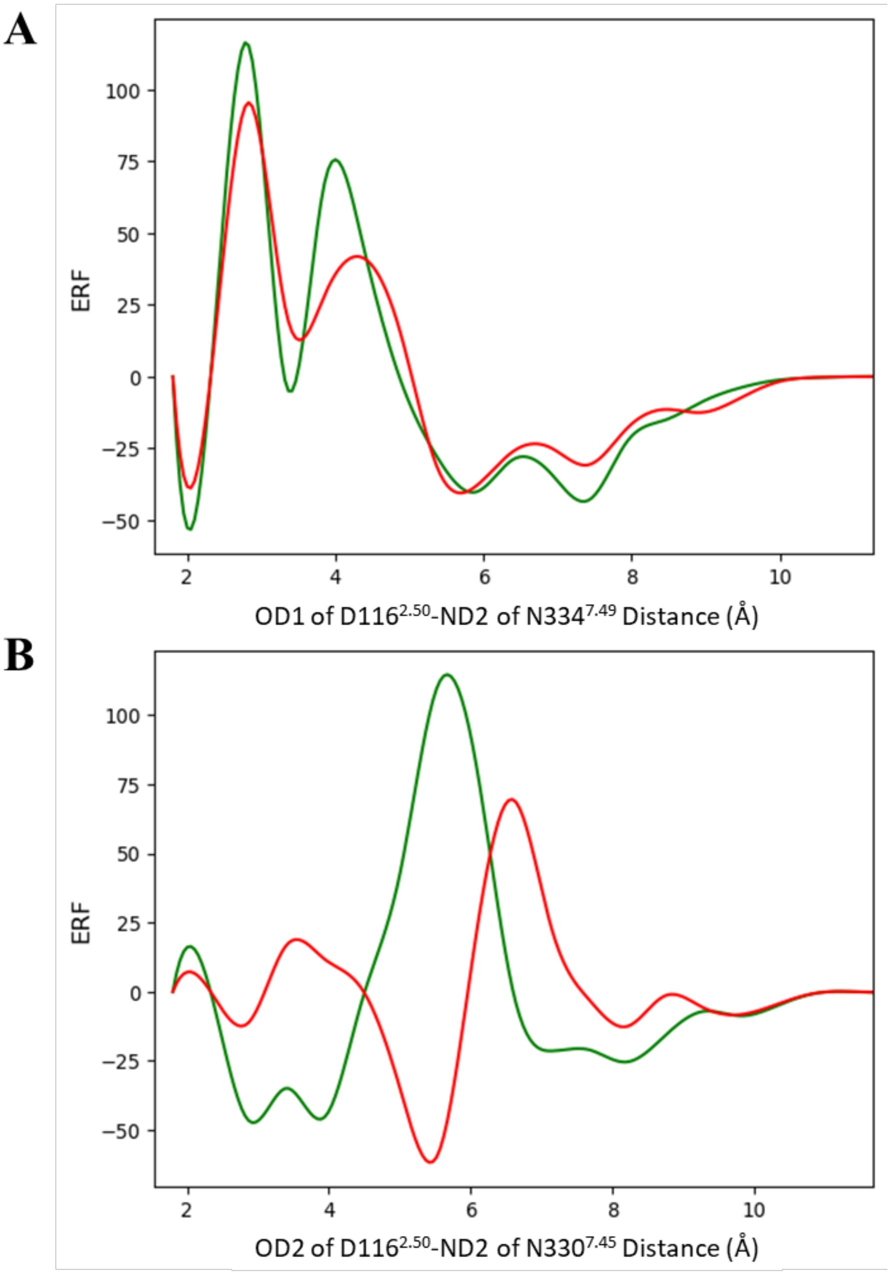

Figure S11 Efficacy response functions of the distance between sodium pocket polar atoms**:** (**A)** OD1 of D116$^{2.50}$ and ND2 of N334$^{7.49}$ and (**B)** OD2 of D116$^{2.50}$ and ND2 of N330$^{7.45}$. The green and red curves correspond to the G protein and β-arrestin-2 efficacy response functions (ERF), respectively.

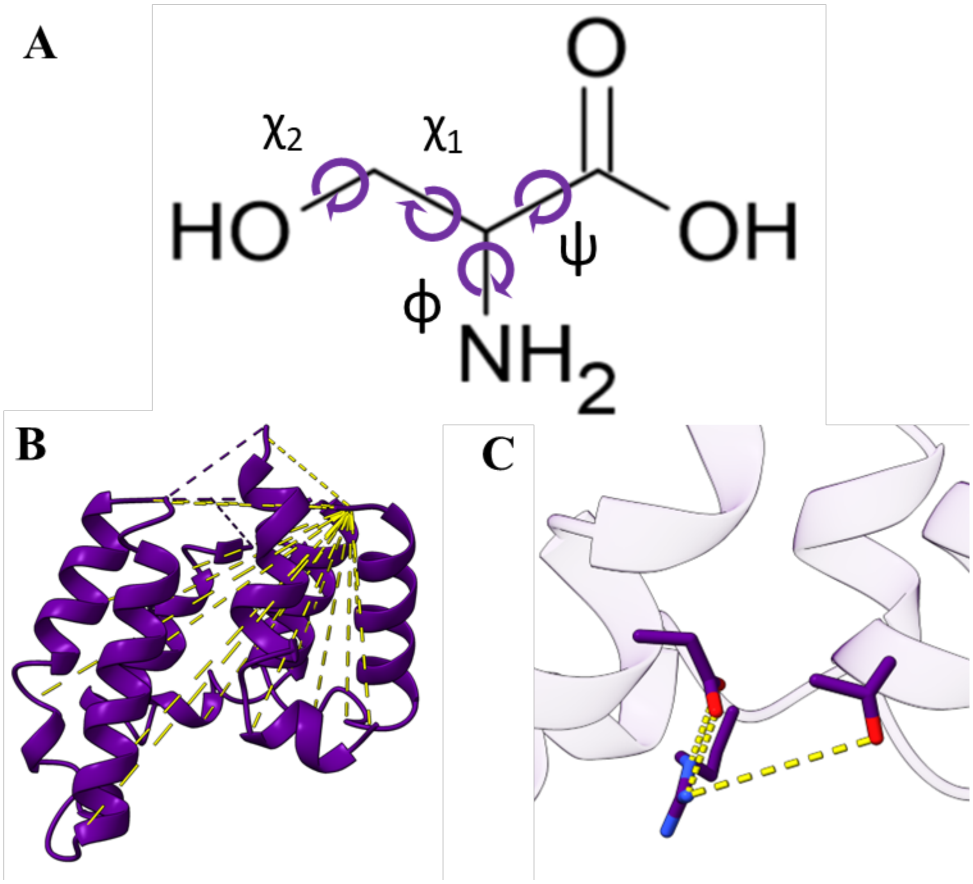

Figure S12 Representations of features used to discretize configurations into conformations: **A)** dihedral angles, **B)** alpha carbon distances, and **C)** distances between polar atoms in the intracellular region of the μOR.

Ligand	Structure	PDBID	G protein E_{max}	β-arr-2 E_{max}	Sources
FH210	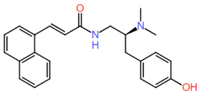	7scg	80 (4)*	18 (2)*	(1)
mitragynine pseudoindoxyl	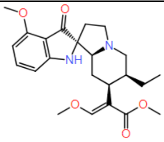	7t2g	81 (4)	0 (0)**	(2)
lofentanil	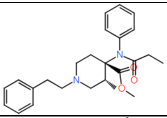	7t2h	115 (2)***	110 (3)	(3)
c6guano	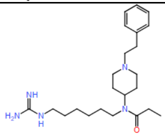	7u2k	137 (2)	19 (1)	(4)
c5guano	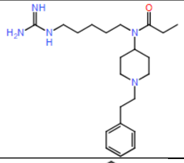	7u2l	104 (3)	54 (3)	(4)
fentanyl	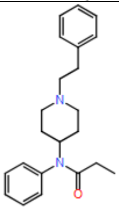	8ef5	92 (4)	60 (2)	(4–8)
morphine	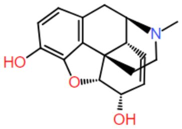	8ef6	97 (4)	28 (3)	(5, 7–10)
TRV130	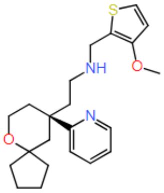	8efb	86 (8)	0 (0)**	(7, 8, 10, 11)
SR10718	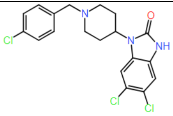	8efl	62 (12)	12 (1)	(5, 7, 8)
PZM21	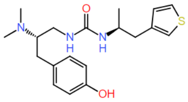	8efo	91 (7)	0 (0)**	(7–10)
DAMGO	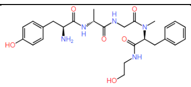	8efq	100	100	

** Converted to DAMGO reference from median experimental reference*
*** Not detectable*
**** Average across multiple Gα subtypes*

Table S1: Summary of structures and efficacy data used for MDS and model training. Emax reported as median Emax (median STD)

Ligand	G protein Recruitment (% of DAMGO)	Uncertainty	Assay	β-arrestin-2 recruitment (% of DAMGO)	Uncertainty	Assay	Source
MP	81	4	cAMP			NR	(2)
TRV130	66	2	cAMP	ND	ND	BRET	(10)
	86	3	cAMP	ND	ND	PathHunter	(11)
	86	5	cAMP	16	3	FRET	(8)
	86	6	cAMP	ND	ND	NanoBit	(7)
PZM21	76	3	cAMP	ND	ND	BRET	(10)
	84	7	cAMP	14	3	BRET	(8)
	97	NR	cAMP	ND	ND	NanoBit	(9)
	112	8	cAMP	ND	ND	NanoBit	(7)
SR17018	105	3	cAMP	10	6	PathHunter	(5)
	62	12	cAMP	12	1	BRET	(8)
	49	12	cAMP	13	5	NanoBit	(7)
Morphine	100	NR	cAMP	14	NR	NanoBit	(9)
	97	1	cAMP	24	1	PathHunter	(5)
	97	4	cAMP	28	3	BRET	(8)
	107	4	cAMP	29	1	NanoBit	(7)
	89	2	cAMP	39	6	BRET	(10)
Fentanyl	97	5	cAMP	60	2	PathHunter	(5)
	NR	NR	NR	59	NR	NanoBit	(6)
	87	4	cAMP	43	4	NanoBit	(7)
	86	2	cAMP	119	3	Tango	(4)
	100	5	cAMP	70	4	NanoBit	(8)
LFT	115	2	BRET	110	3	BRET	(3)
c5guano	104	3	cAMP	54	3	Tango	(4)
c6guano	137	2	cAMP	19	1	Tango	(4)
FH210	80*	4	IP-One	18*	2	PathHunter	(1)

NR Not reported
ND Not detectable
* Converted to DAMGO reference from median experimental reference

Table S2: Experimental cAMP and βarr2 efficacies used to compute the median experimental efficacies for model training.

SI References

1. H. Wang, *et al.*, Structure-Based Evolution of G Protein-Biased μ-Opioid Receptor Agonists. *Angew. Chem. Int. Ed.* **61**, e202200269 (2022).

2. Y. Zhou, *et al.*, Predicted Mode of Binding to and Allosteric Modulation of the μ-Opioid Receptor by Kratom's Alkaloids with Reported Antinociception *In Vivo*. *Biochemistry* **60**, 1420–1429 (2021).

3. Q. Qu, *et al.*, Insights into distinct signaling profiles of the μOR activated by diverse agonists. *Nat. Chem. Biol.* **19**, 423–430 (2023).

4. A. Faouzi, *et al.*, Structure-based design of bitopic ligands for the μ-opioid receptor. *Nature* **613**, 767–774 (2023).

5. C. L. Schmid, *et al.*, Bias Factor and Therapeutic Window Correlate to Predict Safer Opioid Analgesics. *Cell* **171**, 1165-1175.e13 (2017).

6. P. W. De Waal, *et al.*, Molecular mechanisms of fentanyl mediated β-arrestin biased signaling. *PLOS Comput. Biol.* **16**, e1007394 (2020).

7. Y. Zhuang, *et al.*, Molecular recognition of morphine and fentanyl by the human μ-opioid receptor. *Cell* **185**, 4361-4375.e19 (2022).

8. A. Gillis, *et al.*, Low intrinsic efficacy for G protein activation can explain the improved side effect profiles of new opioid agonists. *Sci. Signal.* **13**, eaaz3140 (2020).

9. X. Li, *et al.*, Discovery and Structural Explorations of G-Protein Biased μ-Opioid Receptor Agonists. *ChemMedChem* **17** (2022).

10. A. Manglik, *et al.*, Structure-based discovery of opioid analgesics with reduced side effects. *Nature* **537**, 185–190 (2016).

11. A. M. Gutridge, *et al.*, G protein-biased kratom-alkaloids and synthetic carfentanil-amide opioids as potential treatments for alcohol use disorder. *Br. J. Pharmacol.* **177**, 1497–1513 (2020).
